# Perception of Cats: Assessing the Differences Between Videos and Still Pictures on Adoptability and Associated Characteristics

**DOI:** 10.3389/fvets.2019.00087

**Published:** 2019-03-27

**Authors:** Regina Schoenfeld-Tacher, Lori R. Kogan, Patrick C. Carney

**Affiliations:** ^1^Department of Molecular and Biomedical Sciences, College of Veterinary Medicine, North Carolina State University, Raleigh, NC, United States; ^2^Department of Clinical Sciences, College of Veterinary Medicine and Biomedical Sciences, Colorado State University, Fort Collins, CO, United States; ^3^Cornell University College of Veterinary Medicine, Community Practice Service, Ithaca, NY, United States

**Keywords:** feline, adoption, personality, photograph, video

## Abstract

While animal shelters have made significant progress in reducing the number of euthanized dogs and cats, millions of unclaimed pets are still euthanized every year. Cats, in particular, face bleak prospects, with ~70% of those that enter animal shelters euthanized. Many factors influence potential cat adopters' decisions, including a cat's physical appearance and perceived personality. To explore elements related to the perception of cat personality, this study examined whether videos and pictures highlight different characteristics felt to potentially affect perceived cat adoptability. An online survey was used to assess perceptions regarding videos and pictures of cats. The survey consisted of three adult cats viewed in a short video and as a still picture. Participants were asked to view the media and rate how well these images depicted 12 separate characteristics (from extremely well to not well at all). Respondents were then asked how likely they would be to adopt this cat if they “were in the market to adopt a cat.” A total of 555 surveys were analyzed to answer two questions. The first question was whether cats were perceived as more adoptable when viewed in a still photo or in an action video. A statistically significant difference was found between median photo and video adoption scores for all three cats, with video scores consistently higher than photo scores. The next question was how video footage might alter perception of cats when compared to still photos. For all three cats, the traits “Playful,” “Aggressive,” “Active,” and “Curious” received higher scores when the cats were viewed in videos vs. photos. All of these traits can be associated with active behaviors, best demonstrated via motion. The cats, however, were seen as more “Loving,” “Shy,” “Quiet,” and “Likes to be held” in photos compared to videos. The results suggest that there is an advantage of videos over pictures in perceived adoptability, as determined by response to the question “how likely would you be to adopt this cat,” but this difference is small and likely does not justify additional resources. Exceptions might be for active, outgoing cats in order to highlight these attributes.

## Introduction

Approximately 6–8 million homeless dogs and cats enter the shelter system in the US each year (1). While animal shelters have made significant progress in reducing the number of euthanized dogs and cats, ~3.7 million unclaimed dogs and cats were still estimated to have been euthanized in shelters in 2008 (2). While some of these pets were not adoptable due to health or behavioral reasons, many were euthanized due to a lack of adoptive homes (2). This background provides ample incentive for the exploration of inexpensive and effective methods to increase adoption rates and reduce average length of stay for all species in animal shelters.

Cats, in particular, face unique challenges in animal shelters. A recent U.S. study (2) found that 71% of cats that enter animal shelters are euthanized, compared to 56% of dogs. Even for cats housed in no-kill shelters, timely adoption is still a priority, as the ongoing stress associated with the shelter environment can place cats at increased risk of contracting a disease (3, 4) or developing negative behavioral traits (5). The less time a cat spends in a shelter, the better chances it has to remain healthy and make a successful transition to a new adoptive home. Therefore, factors that influence cat adoption decisions are important topics to more fully understand.

Recent studies have addressed interventions specific to reducing length of stay (LOS) for cats in shelters. Most recently, Janke et al. (6) investigated the impact of a “Capacity for Care” program in Canadian shelters. They found that a combination of lower housing density/cage enrichment, scheduled intake appointments, fast-tracking classification of adoptable cats, reducing adoption restrictions, lowering prices and aiming to have shelters run at or below capacity all helped to reduce average LOS for cats. However, in order for these innovations to be feasible, there needs to a mechanism to promote increased/faster cat adoptions.

Prior research (7) has shown that many pet adopters begin the selection process online, via a site such as petfinder.com. These online profiles play a crucial role in enticing prospective adopters to visit the animals and interact with them in person, evidenced by the fact that 50% of adopters who searched adoptable cat profiles online ultimately adopted the cat they viewed. Even though many other factors such as the animal's age, sex, breed, color and personality are ultimately involved in the decision to adopt a specific animal, this research suggests there is a correlation between the attention an online profile attracts and the animal's LOS in the shelter (7). Animals whose profiles received a greater number of clicks per day had a shorter length of stay in the shelter. While some fixed characteristics, such as age and coat color/length were associated with the number of clicks per day, other conditions which could be controlled by the photographer were not. For example, the animal's position in the profile picture did not affect the number of clicks per day, nor did the inclusion of a human, blanket/bed, or decorative clothing. But photographs that included toys in the setting were clicked on more frequently than those without toys, even when the cat was not directly interacting with the toy (7). The relationship between coat color and perceived cat personality and behavior was further investigated by Delgado et al. (8), who noted that respondents were more likely to perceive orange cats as “friendly” and tri-colored cats as “aloof” and “intolerant.” The relationship between coat color and perceived aggression was further explored by Stelow and et al. (9), with an emphasis on understanding the commonly held perception of calico and tortoiseshell cats being more aggressive. Findings from this study demonstrated that calico, tortoiseshell and torbie cats scored higher on aggression toward humans than all other female cats combined. However, while this information helps explain perceptions, coat color and genetics cannot be altered by shelters seeking prospective adopters. Aside from adding items such as toys to a still photograph, another avenue available for shelters to promote adoptable animals online is to include video clips in their profiles.

The concept of enhancing online profiles was further explored by Pyzer et al. (10), who investigated the impact of video footage vs. still photographs on perception of behavioral traits and adoptability for dogs. These authors hypothesized that videos would allow for fuller portrayal of such traits as sociability, obedience and friendliness than still images. They gathered video footage and photographs of 4 dogs, and used an online questionnaire to investigate the extent to which viewers perceived the dogs as having desirable (trainable, intelligent, friendly, gentle, playful and obedient) and undesirable (dominant, aggressive, assertive, unsociable, hyperactive, and fearful) behavioral traits. There were significant differences in how each dog was perceived via video vs. still image. Dogs seen in videos were perceived as more trainable, intelligent, friendly and gentle, and less dominant, aggressive and unsociable than they were in photographs. Thus, Pyzer et al. concluded that video might be a more beneficial form of advertising dogs than still photographs.

Isgate and Couchman (11) conducted an in-depth study about the characteristics of photographs that make dogs appear more adoptable. The authors used an eye-tracking device to study gaze patterns of 50 undergraduate students, as they viewed photographs of four different dogs, a Doberman, Golden Retriever, Pit Bull and Rottweiler. Each dog was shown in four standardized poses—standing alone, sitting alone, sitting with handler, and walking on leash. Results showed that the breed, pose and facial features of each dog affected areas of attention on the photograph and perceived personality. The authors concluded that potential adopters readily attributed personality and behavioral traits such as friendliness and aggressiveness to dogs in photographs, and these perceptions influenced decisions about adoptability.

This study will examine a similar intervention for promoting adoption of cats.

### Factors Influencing Adoption of Cats

There is contradictory evidence surrounding the influence of physical characteristics on perceived adoptability of cats. Some studies have shown that coat color plays a role in adoption decisions (12–15), while other studies such as Sinn (16) have found little evidence to support the influence of characteristics such as coat length and color in selecting a cat for adoption.

Behavioral and environmental characteristics may play a much larger role in decisions to adopt. A cat's perceived friendliness and desire to play can greatly influence adopters' decisions. In a study involving 43 cats adopted from a local shelter, Kry and Casey (17) found that temperament was the highest ranked reason for choosing a cat for adoption, while the physical appearance of the cat or kennel did not impact adoption decisions. Weiss et al. (18) surveyed a total of 1,491 adopters across five animal shelters. Within this group, 26.9% of cat adopters cited behavior as the single most important reason they selected their pet. Based on these findings, Weiss et al. (18) suggested that providing a toy for potential adopters to use when viewing a cat, and/or training cats to come to the front of their cage could be helpful interventions to highlight cats' desirable behaviors. Sinn (16) found that 81% of adopters rated “friendliness toward me” as very important when deciding to adopt a cat and 68% rated playfulness as a desirable characteristic. Environmental factors associated with the adopter-cat meeting were also important, as 95% of adopters reported that the opportunity to interact directly with the cat played a role in their decision. Additionally, 61% of respondents reported that it was important to be able to play with the cat or see it play with toys, further reinforcing the potential impact of a cat's playfulness and activity level on perceived adoptability. These trends continue to be supported in the current literature.

Southland et al. (19) surveyed 130 visitors to an adoption center, including those who did not adopt a pet during their visit, about the characteristics they were looking for in a prospective pet. Among respondents seeking to adopt a cat, the most commonly selected traits were: affectionate, friendly and in good health. The need for a potential pet to be affectionate was more important to prospective adopters of cats than those seeking a dog. When responses were further narrowed down to compare participants who adopted a cat during the study with those who did not, the most common reason for adopting a particular pet was that the animal was affectionate, friendly and playful. Among non-adopters, an animal's lack of reaction to them was the most common reason given for not adopting a pet on that particular day. The authors postulated that an animal's behavior in and outside the kennel can be seen as indicators of its personality, and thus influence adoption decisions.

A pilot study of cat adopters at a limited-admission, no-kill shelter in the Western US, conducted by the authors of this paper (results not published) provided empirical information to support the published research. Personality was most frequently cited as a contributing factor for selecting a specific adult cat, as reported by 84% of participants. Other commonly reported factors were behavior, age, and appearance. When queried about specific behaviors influencing their choice, adopters most frequently selected “the cat was friendly with me/my family” as an influence, followed by “the cat was purring,” “the cat allowed me/my family to hold it” and “the cat rubbed on my hand or the cage doors.”

### Cat Personality Analysis

Extensive research has been conducted in cat personality, resulting in a variety of models. Most studies are based on Feaver's et al. (20) finding of three major factors of personality: alert, sociable and equable. Siegford et al. (21) developed broad temperament tests designed to measure a cat's sociability, aggressiveness and adaptability, in order to help match the animal with a suitable adoptive home. These earlier studies paved the way for development of the Feline-ality™ test (22, 23), which is designed to match cats with potential adopters based on the cat's expected behavior in a home setting and the adopter's expectations. Bradshaw (24) and Gartner and Weiss (25) postulated other three-factor models, based on different aspects of personality. However, current trends favor personality models with a greater number of factors.

Ha and Ha (26) conducted a meta-analysis of the three-factor models and utilized common trends across these studies as a basis to develop a web-based survey for cat owners. Their results lent support to a five-factor model, based on the following: cat social (social with other cats), active, human non-social, human aggressive and intense (main trait is vocal). Litchfield et al. (27) also support a five-factor model of cat personality with the following labels: neuroticism, extraversion, dominance, impulsiveness, and agreeableness. These factors translate into observable behaviors in cats, and the authors point out that high scores on agreeableness are associated with cats perceived as “friendly” and “happy,” with these cats having the highest likelihood of adoption. Similar results were reported by Bennet et al. (28), who examined adjectives that best describe cat personality. After conducting a study of 416 adult cat owners, they identified six personality dimensions: playfulness, nervousness, amiability, dominance, demandingness and gullibility. Together, the six factors studied by Bennet (28) explained 56.08% of the total variance in reported cat personality. The vast amount of research in the area of cat personality, including a comprehensive review by Gartner (29) points to the fact that there are many ways to measure cat personality, and that this subject still needs further investigation and standardization of methods.

To further explore elements related to the perception of cat personality, as determined through online viewing, we designed a study to investigate if videos and pictures highlight different characteristics felt to potentially affect perceived cat adoptability. Our research questions were:

How do videos vs. still pictures of the same cat alter their perceived adoptability?
How do videos vs. still pictures of the same cat alter their perceived traits and which of these traits best predict perceived adoptability?

Our hypothesis was that videos of cats playing/active would positively influence viewers' perception of the cats, making them appear more adoptable. We also hypothesized that several feline traits associated with increased likelihood to adopt would be more prevalent in the videos compared to still pictures.

## Materials and Methods

### Study Design

An online survey was created in Qualtrics to assess perceptions regarding videos and pictures of cats. The survey was designed, reviewed, and tested by the co-investigators and their colleagues at Colorado State University (CSU) and North Carolina State University (NCSU) who provided feedback on content, navigability, survey questions and choices, and overall questionnaire design. The survey originated from CSU and received approval from the Institutional Review Board at CSU. Participants were recruited via Amazon Mechanical Turk, from April 10, 2018 to April 26, 2018. Created in 2005, Mechanical Turk is a crowdsourcing online labor market that coordinates the supply and the demand of cognitive tasks (30). This Internet sample is reliable, older, and more diverse than typical college student samples (31–33). All data were collected anonymously. The survey began by asking participants to indicate if they lived in the United States. Those not residing in the US were eliminated from analysis.

Three adult cats were included for analysis and each cat was viewed in two conditions: a still picture taken of the cat inside its cage, and ~10-s video in which the cat interacted with a toy inside of its cage. The length of the videos was chosen in order to ensure the survey would load quickly for participants. All the cats were spayed, adult females. At the time the photos/video were taken, Georgia was a 1.5 year old gray tabby with white, Ginger a 5 year old orange tabby, and Pretty a 1.5 year old tortoiseshell. The particular cats were chosen because their coloring made them easy to photograph and prior research (7, 12, 13, 15) has shown that bicolor and tabby cats are more adoptable than their all-black counterparts. All were successfully adopted from the shelter prior to the launch of the survey. The photographs (see Supplementary Figure 1) and videos (see Supplementary Videos 1–3) in this study were obtained from a local cat shelter, and taken by shelter staff members for the purpose of this study. Respondents were exposed to three cat pictures, and three cat videos. The order of presentation was fully randomized by the survey software.

The survey was comprised of demographic questions and then questions about each picture/video. Respondents were first asked to indicate demographics including sex, educational level, geographical area (urban, rural, suburban) of residence, marital status, number of children under 18 living at home, age, number of cats currently owned, number of cats ever owned, and feelings about cats in general (e.g., really dislike to love cats). Each of the 6 cat/media pairings were presented in random order, and participants were asked to rate how well the photograph or video described each of the 12 personality traits under study. These characteristics included: playful, talkative, loving, friendly, shy, aggressive, sweet, quiet, demanding, likes to be held, active, and curious. These ratings were based on a 5-point Likert scale. The anchors were: extremely well, very well, moderately well, slightly well, not well at all. Respondents were then asked how likely they would be to adopt this cat if they “were in the market to adopt a cat.” The response options were: Extremely likely, somewhat likely, neither likely nor unlikely, somewhat unlikely, extremely unlikely, I would never adopt any cat. This data was collected for each cat/condition. No additional information (sex, age, etc.) was provided about the cats. A free text area was provided at the end of the survey to allow participants to make additional comments. The amount of time respondents spent viewing the videos/photographs was not tracked.

### Statistical Analysis

The first outcome of interest was defined as the difference between the video adoption score and the photograph adoption score. A secondary outcome was defined as a change in adoption status, which was a binary outcome indicating whether the respondent's decision to adopt a given cat changed between the photo and video assessments. For the purposes of this outcome, video or photo adoption scores of 1–3 were grouped in a “would not adopt” category, while scores of 4–5 formed a “would adopt” category. Respondents who answered, “would never adopt a cat” at any point in the survey were not included in the analysis.

Analysis was first conducted separately for each of the three cats. Normality of the outcome was assessed graphically via histograms and formally via the Shapiro–Wilk test. The sign test was employed to evaluate the difference in video and photo scores.

The data for all three cats were then analyzed together. A Kruskal–Wallis test was used to assess whether the primary outcome differed significantly between the three cats. Normality of the outcome was again assessed via examination of a histogram and the Shapiro–Wilk test. Candidate predictors were evaluated for collinearity via linear regression and calculation of the tolerance and variance inflation factors. A mixed effects model was then fitted with all candidate predictors (sex, age, education level, location [urban, suburban, rural], marital status, number of children, sentiment toward cats, total number of cats owned, and number of cats currently owned) entered as fixed effects, and with cat and respondent as random effects. *P*-values for fixed effects were determined by likelihood ratio tests of the fully specified model vs. the model with the predictor of interest removed. Predictors with *p* < 0.2 were retained in the final model.

To investigate the impact of twelve specific descriptors (playful, talkative, loving, friendly, shy, aggressive, sweet, quiet, demanding, likes to be held, active, and curious) on adoption scores, ordinal logistic regression was performed for each individual cat and for all three cats combined, with photo adoption score and video adoption score as the dependent variables. Models were fit using backwards selection with all variables *p* < 0.2 retained. In the model incorporating all three cats, for ease of interpretation of the results the retained variables were then investigated for inclusion as linear, rather than ordinal, predictors by fitting a linear model with each variable entered twice, once as an ordinal and once as a linear predictor; those with type III sums of squares with *p* ≥ 0.05 were entered as linear predictors in the final model, while those with *p* < 0.05 were retained as ordinal variables. Cat and respondent were modeled as random effects in the final model.

The correlation between scores for the 12 predictors assessed via photo vs. video was examined two ways. First, Kendall's tau-b was calculated for each photo-video pairing for all three cats combined. Second, Bowker's test for symmetry was applied for each individual cat and for all cats combined.

Data manipulation was performed using the Pandas data analysis library for the Python programming language, as implemented in the Enthought Canopy environment. Statistical analyses were carried out using SAS version 9.4.

## Results

### Respondent Characteristics

A total of 576 surveys were submitted, of which 21 (3.6%) indicated on at least one question that the respondent would never adopt a cat and were subsequently omitted from the analysis. The excluded data came from 10 male and 11 female respondents. Among these 21 respondents, 9 reported really disliking cats, 7 didn't care for them, and only 2 stated they loved cats. Of the remaining 555 respondents, 201 (36.2%) were male, 352 (63.4%) were female, and 2 (0.4%) identified as “other”; 167 (30.1%) indicated urban place of residence, 272 (49.0%) indicated suburban, and 116 (20.9%) indicated rural. The most common age category was 31–40 (148 respondents, 26.7%); 96 respondents (17.3%) were 18–25, 109 (19.6%) were 26–30, 93 (16.8%) were 41–50, 67 (12.1%) were 51–60, 36 (6.5%) were 61–70, and 6 (1.1%) were older than 70 years of age. Fifty-three (9.5%) had a high school diploma or GED as the highest level of educational attainment, 223 (40.2%) had some college, 198 (35.7%) had a 4-year degree, 75 (13.5%) had a graduate degree, and 6 (1.1%) selected other or declined to answer. For marital status, the most common categories were married, with 265 respondents (47.8%), and single, with 225 (40.5%), followed by divorced (46 respondents, 8.3%), widowed (14 respondents, 2.5%), and other (5 respondents, 0.9%). Three hundred sixty-nine (66.5%) had no children; for those with children, the median number of children was 2 (IQR 1), with a range of 1–10. A full breakdown of the data for all respondents compared to those retained in the study is available in Table 1.

**Table 1:** Demographic characteristics of all respondents, and those remaining in the study after excluding responses from those that would “never adopt a cat.”

	**All respondents (*****n*** **= 576)**		**Retained in study (*****n*** **= 555)**	
	**Frequency**	**Percent**	**Frequency**	**Percent**
**SEX**				
Male	211	36.6	201	36.2
Female	363	63.0	352	63.4
Other/NA	2	0.3	2	0.4
**AGE**				
18–25	104	18.1	96	17.3
26–30	112	19.4	109	19.6
31–40	152	26.4	148	26.7
41–50	97	16.8	93	16.8
51–60	69	12.0	67	12.1
61–70	36	6.3	36	6.5
Over 70	6	1.0	6	1.1
**HIGHEST LEVEL OF EDUCATION COMPLETED**				
High school/GED	57	9.9	53	9.5
Some college or associate's degree	230	39.9	223	40.2
Bachelor's degree	205	35.6	198	35.7
Graduate degree	77	13.4	75	13.5
Other	2	0.3	2	0.4
**AREA OF RESIDENCE**				
Urban	170	29.5	167	30.1
Rural	121	21.0	116	20.9
Suburban	285	49.5	272	49.0
**MARITAL STATUS**				
Single	235	40.8	225	40.5
Divorced	46	8.0	46	8.3
Widowed	14	2.4	14	2.5
Married/Partnered	276	47.9	265	47.7
Other	5	0.9	5	0.9
**NUMBER OF CHILDREN (UNDER 18 YEARS) LIVING IN HOME**				
None	379	65.8	369	66.5
1	80	13.9	75	13.5
2	75	13.0	72	13.0
3	25	4.3	25	4.5
4	7	1.2	6	1.1
5 or more	10	1.7	8	1.5

When queried about sentiment toward cats, the majority (333 respondents, 60.0%) reported loving cats, 145 (26.1%) reported liking cats, 62 (11.2%) were okay with cats, 13 (2.3%) did not really care for cats, and 2 (0.4%) reported really disliking cats. The median number of cats currently in the respondent's household was 1 (IQR 2), and the median cumulative number of cats owned by the respondents was 2 (IQR 4). Two hundred one respondents (36.2%) reported having no cat currently in the household; for those with at least one cat in the household, the median number of cats was 1 (IQR 1). Seventy-nine respondents (14.2%) reported never having owned a cat; for those having owned at least one cat, the median cumulative number of cats owned was 3 (IQR 3). A full breakdown of cat ownership status and sentiment towards cats voiced by all respondents compared to those retained in the study is available in Table 2.

**Table 2:** Cat ownership status and sentiment toward cats, for all respondents as compared to those retained in study.

	**All respondents (*****n*** **= 576)**		**Retained in study (*****n*** **= 555)**	
	**Frequency**	**Percent**	**Frequency**	**Percent**
**NUMBER OF CATS CURRENTLY OWNED**				
None	220	38.2	201	36.2
1	195	33.9	195	35.1
2	92	16.0	92	16.6
3	33	5.7	31	5.6
4	20	3.5	20	3.6
5 or more	16	2.6	16	3.0
				
**NUMBER OF CATS OWNED AS AN ADULT, INCLUDING CURRENT CATS**				
None	96	16.7	79	14.2
1	106	18.4	106	19.1
2	102	17.7	101	18.2
3	69	12.0	69	12.4
4	54	9.4	54	9.7
5	33	5.7	32	5.8
6	32	5.6	31	5.6
7	19	3.3	18	3.2
8	12	2.1	12	2.2
9	6	1.0	6	1.1
10 or more	47	8.2	47	8.4
				
**FEELINGS ABOUT CATS IN GENERAL**				
I love cats	335	58.2	333	60.0
I like cats	148	25.7	145	26.1
Cats are OK	62	10.8	62	11.2
I don't really care for cats	20	3.5	13	2.3
I really dislike cats	11	1.8	2	0.4

### Individual Cat Analysis

The distributions of the differences between photo and video scores for all three cats were found to be non-normal (Shapiro–Wilkes test for normality, *p* < 0.0001 for all three) due to mild to moderate negative skewness. All three cats had a median difference in score of 0 (IQR 1), and the means for all three fell between −0.18 and −0.30, consistent with the negative skewness. The sign test found a statistically significant difference between the median photo and video scores for all three cats (*p* < 0.0001 for all three), with video scores being higher than photo scores.

**Figure 1:**
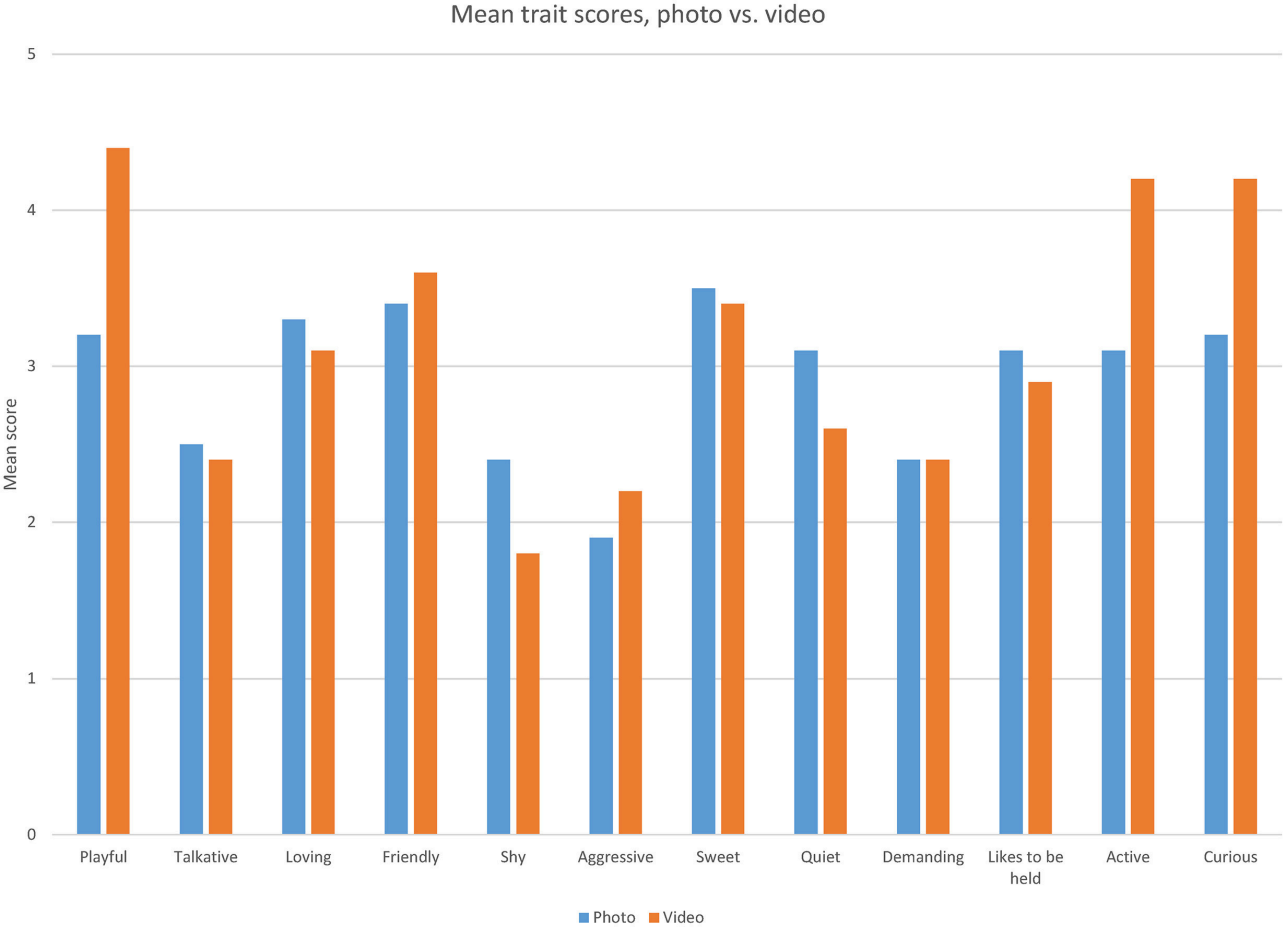
Mean scores for each of the studied personality traits, as reported for the cats seen in photographs vs. videos.

Among twelve specific descriptors of the photos and videos, “Shy” was the lowest-scored for both photos and videos for each of the three cats (median 1 or 2). “Aggressive” and “Demanding” were similarly consistently scored low. “Sweet” was the highest-scoring descriptor (median 4) for all three cats when assessed via photo; “Playful” was the highest-scoring descriptor (median 4) for all three assessed via video (see Figure 1).

**Figure 2:**
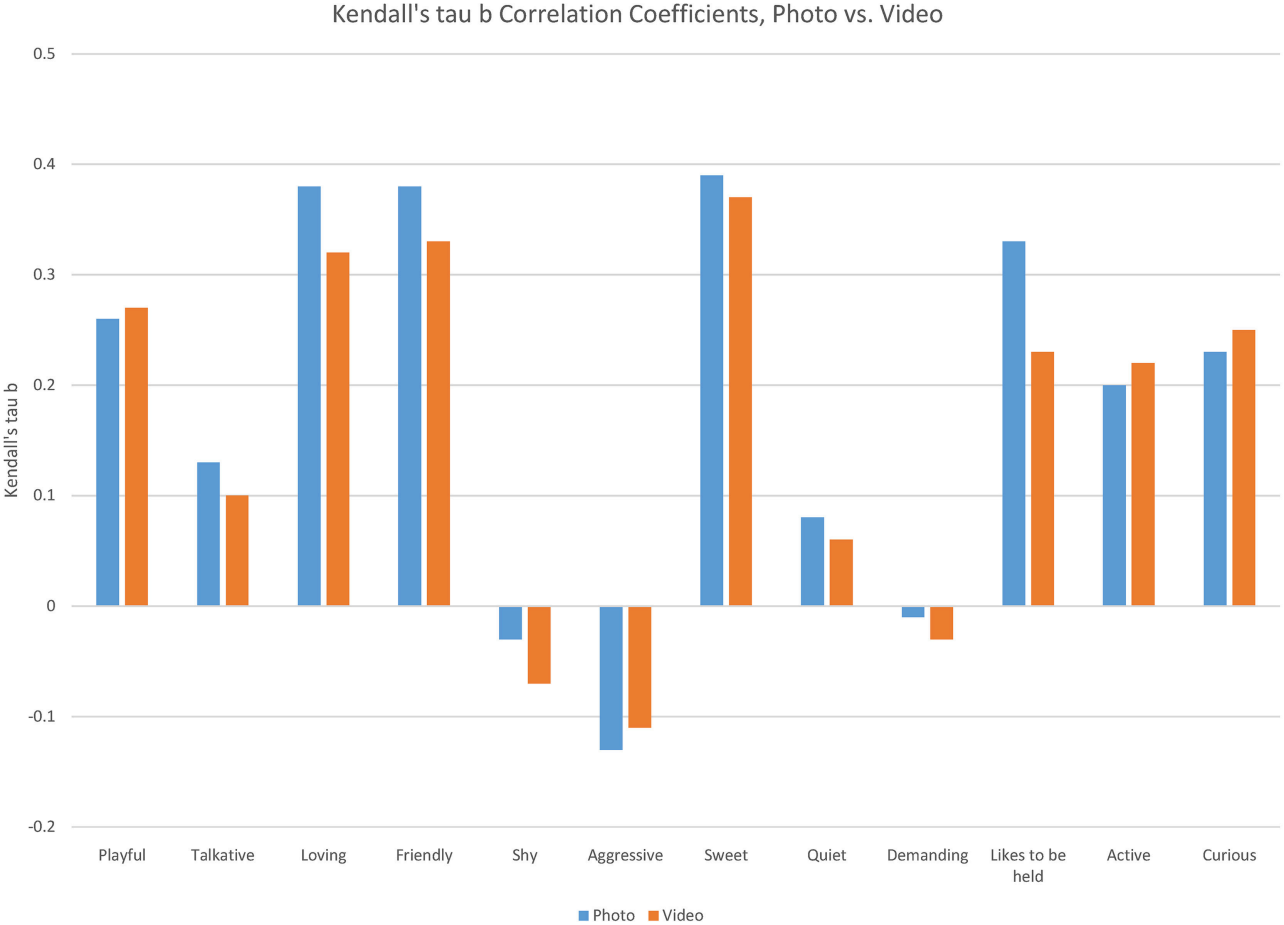
Kendall's tau B correlation coefficients, as observed for each of the personality traits rated in photographs vs. videos.

For photos, “Aggressive” was the only descriptor that was negatively correlated with adoption score in bivariate analysis for all three cats (Kendall's tau-b range −0.151 to −0.091; *p* = 0.0155 to < 0.0001); “Demanding” and “Shy” showed no significant association (all *p* > 0.0975). “Quiet” showed no correlation with photo adoption score in two of the three cats; in the third (Ginger, orange tabby), it had a slight positive correlation (Kendall's tau-b 0.127, *p* = 0.0005). “Play,” “Talkative,” “Love,” “Friendly,” “Sweet,” “Likes to be held,” “Active,” and “Curious” were all positively correlated with photo adoption score (*p* all < 0.05) in all three cats, with “Love,” “Sweet,” and “Friendly” having the strongest correlation in all three cats (Kendall's tau-b range 0.367–0.400) (Figure 2).

For videos of all three cats, “Aggressive” was negatively correlated with adoption score in bivariate analysis for two of the three cats, and had no significant correlation in the third (Pretty, tortoiseshell) (Kendall's tau-b range−0.194 to−0.053, *p* < 0.0001 to *p* = 0.164). “Quiet” and “Demanding” had no significant correlation for any of the cats (all *p* > 0.0517). Identical to photo adoption score, “Playful,” “Talkative,” “Love,” “Friendly,” “Sweet,” “Likes to be held,” “Active,” and “Curious” were all positively correlated with video adoption scores (*p* all < 0.05) in all three cats, with “Love,” “Sweet,” and “Friendly” having the strongest correlation in all three (Kendall's tau-b range 0.285–0.4).

### Combined Analysis

Adoption rating scores for the photos and videos were found to have a moderately strong positive correlation (Kendall's tau-b 0.58, 95% confidence interval 0.54–0.61). In 60.2% of the photo-video score comparisons the respondents' scores did not change. In 29.3% of comparisons the respondents scored the cat higher on video than on photos, whereas in the remaining 10.5% the photos were scored higher than video.

When assessing the change in adopting status, respondents were significantly more likely to adopt based on video (McNemar's test statistic 84.05, 1 degree of freedom, *p* < 0.0001). Of 1665 photo-video score comparisons, 1,313 were concordant (no change in adoption likelihood), 90 were discordant in favor of photos, and 262 were discordant in favor of video. Descriptors with a trend toward higher video scores included “Friendly” (mild), “Aggressive” (mild to moderate), “Curious” (moderate to strong), and “Active” and “Playful” (strong). Descriptors with a trend toward higher picture scores included “Sweet” (strong), “Likes to be held” (mild to moderate), “Loving (mild to moderate) and “Quiet” (mild).

## Discussion

This study was designed to shed light on two questions. The first question is whether cats were perceived as more adoptable when viewed in a still photo or in an action video. To answer this question, participants rated adoptability of three cats, all of which were displayed as a picture or short video. A statistically significant difference was found between median photo and video adoption scores for all three cats, with video scores consistently higher than photo scores. For these photo-video adoption score comparisons, 15.7% of participants rated the cats as more adoptable when viewing videos, 78.9% did not favor videos or pictures when determining adoption scores, and 5.4% rated the cats as more adoptable when viewing photos.

Even though respondents indicated higher likelihood of adopting when viewing videos vs. photos, this difference was relatively small. On a 5-point Likert scale, the differences were between 0.18 and −0.30; or stated differently, ~a 1/5 to a 1/3 point difference between video and photo. Thus, the answer to the first question is yes, there is a difference between perceived adoptability of cats when viewing them as a still picture vs. a video, with videos offering a small advantage. This finding is consistent with Pyzer et al.'s (10) study showing that video footage tended to improve perception of positive behavioral traits, as compared to still images.

The next issue to consider is how viewing video footage instead of still images might lead to the observed changes in perception. Are there specific traits seen in videos (or pictures) that influence perceived adoptability? Before we can ask what differences there are between videos and pictures (besides the obvious fact that videos can display movement and photos do not), the first question to determine is what characteristics influence adoptability, regardless of viewing format. The foundational question is therefore, what traits appear to influence the perception of adoptability. The answer to this question can add meaning to how the perception of these traits change when viewed via picture vs. video.

The existing literature (25–29) on cat personality models was reviewed, and the 12 most commonly studied traits, were used to develop the survey questionnaire. These traits included: playful, talkative, loving, friendly, shy, aggressive, sweet, quiet, demanding, likes to be held, active, and curious. For both photos and videos, the traits “Playful,” “Talkative,” “Loving,” “Friendly,” “Sweet,” “Likes to be held,” “Active,” and “Curious” were all positively correlated with adoption scores. “Loving,” “Sweet,” and “Friendly” had the strongest correlations with adoption ratings.

The fact that these eight traits correlated with adoption scores aids in the exploration of how these traits are interpreted differently based on whether a cat is viewed in a video or a picture.

For all three cats, the traits “Playful,” “Aggressive,” “Active,” and “Curious” received higher scores when the cats were viewed in videos vs. photos. All of these traits can be associated with active behaviors, best demonstrated via motion. Yet, the cats were seen as more “Loving,” “Shy,” “Quiet,” and “Likes to be held” in photos compared to videos. This is not surprising, because these traits are more abstract and difficult to observe (unless of course a cat is photographed while being held). Perception of a cat as shy or quiet might require the viewer to make their own interpretation of the image, and this is perhaps easier to do with a still photograph, as demonstrated in Isgate and Couchman's (11) study of dog images. Traits showing the greatest difference between photo and video scores, as reflected in moderate to strong trends in all three cats, were “Playful,” “Shy,” “Active,” and “Curious.” Therefore, when assessing the characteristics associated with adoptability, there is no clear advantage of video over pictures or vice versa. The best media to use depends on which characteristic is being assessed. The more action-oriented traits are scored higher in videos and the more sedentary traits are scored higher in photos. Due to the study design, assessment of the sedentary traits likely required additional imagination/projection from respondents. Since there was no sound associated with the videos or photos, viewers would have to deduce for themselves whether the cats were vocalizing or not. The traits “loving,” “shy” and “likes to be held” all imply a relationship with another animal or human. Since the cats were depicted alone in a cage, in the videos/photos, viewers would also have to infer these characteristics. It might have been easier for respondents to add their own interpretations to photographs vs. videos. Both sets of traits are correlated with adoption scores; yet perhaps not equally for all potential adopters. Some studies, for example, have found that potential adopters appear to be influenced by activity level and perception of playfulness (10, 19, 34, 35).

We have learned there is an advantage of videos over pictures in perceived adoptability, but this difference is small and likely does not justify an outpouring of resources if a shelter does not already have the equipment and personnel to video cats.

It is important to note that only some of the traits that correlate with adoptability are rated higher in videos. These traits tend to be more action oriented. Given the fact that videos typically display movement, this makes sense. Cats are seen as more “Playful,” “Active,” and “Curious” when presented via video. All these are positive traits that correlate with adoption ratings. Cats are also seen as more aggressive in videos, and this characteristic is negatively correlated with adoption, so care should be taken to minimize video footage that might make cats appear aggressive. Given these active attributes, it might behoove shelters to choose more active, outgoing cats to showcase in adoption videos, given that these cats have traits that can be highlighted in videos. Yet, some traits are demonstrated better in photos, and these traits are more sedentary (“Loving,” “Shy,” “Quiet,” and “Likes to be held”). For the quieter, lap-cats, photos, therefore, might be the appropriate tool. Another aspect to consider is the color of the cat. Given the existing literature (8, 9) documenting the effect of coat color on perceived personality, we would have expected the orange cat (Ginger) to be perceived as more friendly overall. When her scores were compared for the photo and video conditions, being rated as quieter seemed to increase her potential adoptability. This leads us to speculate as to whether photographs may be a better vehicle for showcasing orange cats. Tortoiseshell cats such as Pretty have been perceived as more aloof and intolerant (8), and have shown increased aggression levels toward humans (9). In this study, there was a negative correlation between perceived aggressions and adoptability, for the gray/white tabby and the orange cats. Yet, perceived aggression did not play a role in adoptability ratings for the tortoiseshell. This opens the door to speculation on possible over-compensation and baseline assumptions that could have been made about each cat due to their coloring. However, these assumptions would not affect the results of this study, because each cat served as their own control for the photo vs. video ratings.

Some shelters might have the resources to obtain both pictures and videos of their cats. In that case, it might be helpful to determine if potential adopters are looking for an active cat or a “lap cat.” For those hoping to adopt an active playful cat, a video is likely to be more effective. For those potential owners looking for a quiet, loving cat, it is suggested they be shown pictures.

This study offers some insights into the question of whether to invest resources in videotaping cats as an adoption-enhancing tool, and the answer appears to be, due to the small effect of videos over pictures, that shelters might be better advised to allocate their limited resources elsewhere. If shelters do have the capability to record video clips, these may prove especially useful if strategically used to highlight the more active, playful cats.

Limitations of this study included the fact that cats were photographed and videotaped inside of their cages. This was done in order to standardize the setting, and minimize interference from other people and animals. We chose not to feature humans in the media, in order to make it easier for potential adopters to imagine owning the pet, and bonding with the cat. The cats used in this study were all adult females, with vibrant coloring. Prior research (13) has demonstrated a relationship between coat color and length of stay in a shelter, so coat color may have influenced the results of this study. However, this risk was minimized by having each cat serve as its own control, and comparing perceived adoptability of each individual in a photograph vs. a video. This study design also minimized any potential impact of cat age on perceived adoptability, because the photograph of each cat was compared to a video of the same cat taken on the same day. This study design also reduced the risk of social desirability bias, as the outcome under study was the difference in ratings for a particular cat. Other precautions taken to reduce social desirability bias included collecting data anonymously, and wording the questionnaire in a neutral manner (36, 37). Moreover, social desirability bias is of greatest concern when respondents are being asked extremely personal questions about topics that carry strong social connotations, either positive or negative; it is unlikely that questions asking for a respondent's opinion about traits in a cat to which he or she has no prior connection are likely to strongly evoke social desirability bias (38, 39).

Data collection via Amazon Mechanical Turk may have resulted in a respondent pool that is slightly younger than the US population as a whole. While this may limit the applicability of study results to older adopters, it also strengthens the generalizability of the results to the target audience. Millennials are the largest segment of pet owners, accounting for 35% of all pet owners (40). This group is significantly more likely than all other generations to have learned a cat was available for adoption via the Internet (40), making them the most likely group to be influenced by photographs vs. videos of adoptable cats.

Since the goal of the study was to examine the effect of presentation media on the preferences of potential cat adopters, we chose to retain participants who do not currently own a cat, or have never owned a cat as in this study. They are an important segment of our target population, because the American Pet Products Association study (40) showed that 22% of cat owners have not owned a previous pet. And, among current cat owners, only 27% adopted their new pet while they still had their previous pet. Thus, this is an important demographic segment to consider when exploring mechanisms shelters can use to encourage new adoptions.

Given the results of this small-scale study, additional investigation is warranted to explore other potential impacts of video footage on cat adoptions. The next step is to investigate whether video footage depicting cats engaging in active play and other activities outside of their cages has a greater influence on adoption decisions, as compared to photographs of cats outside of their cages. This builds on research by Protopopva and Wynne (41), showing that humans were more likely to adopt dogs that would lay closer to them during shelter visits, and respond to their attempts to initiate play. Another aspect to investigate would be whether the observed trends hold true for cats of different colors/coat lengths and ages. Depending on the results of this study, further investigations could explore whether video footage highlighting active, playful behaviors can play a role in promoting adoption of senior cats. Video could also be used to show the playful, loving aspects of shy cats, who may initially hide from strangers in a shelter setting.

## Data Availability

The datasets generated for this study are available on request to the corresponding author.

## Author Contributions

LK and RS-T conceived the study, conducted the research, and wrote the manuscript. PC conducted analyses and helped prepare the manuscript.

### Conflict of Interest Statement

The authors declare that the research was conducted in the absence of any commercial or financial relationships that could be construed as a potential conflict of interest.

## Supplementary Material

Supplementary Figure 1 Still photographs used in study.

Supplementary Video 1 Video of cat 1—Georgia.

Supplementary Video 2 Video of cat 2—Ginger.

Supplementary Video 3 Video of cat 3—Pretty.
