# Supplementary figures and images for: Perception of Cats: Assessing the Differences Between Videos and Still Pictures on Adoptability and Associated Characteristics
Source: Front Vet Sci. 2019 Mar 27;6:87. doi: 10.3389/fvets.2019.00087 (PMC6445959; doi:10.3389/fvets.2019.00087)

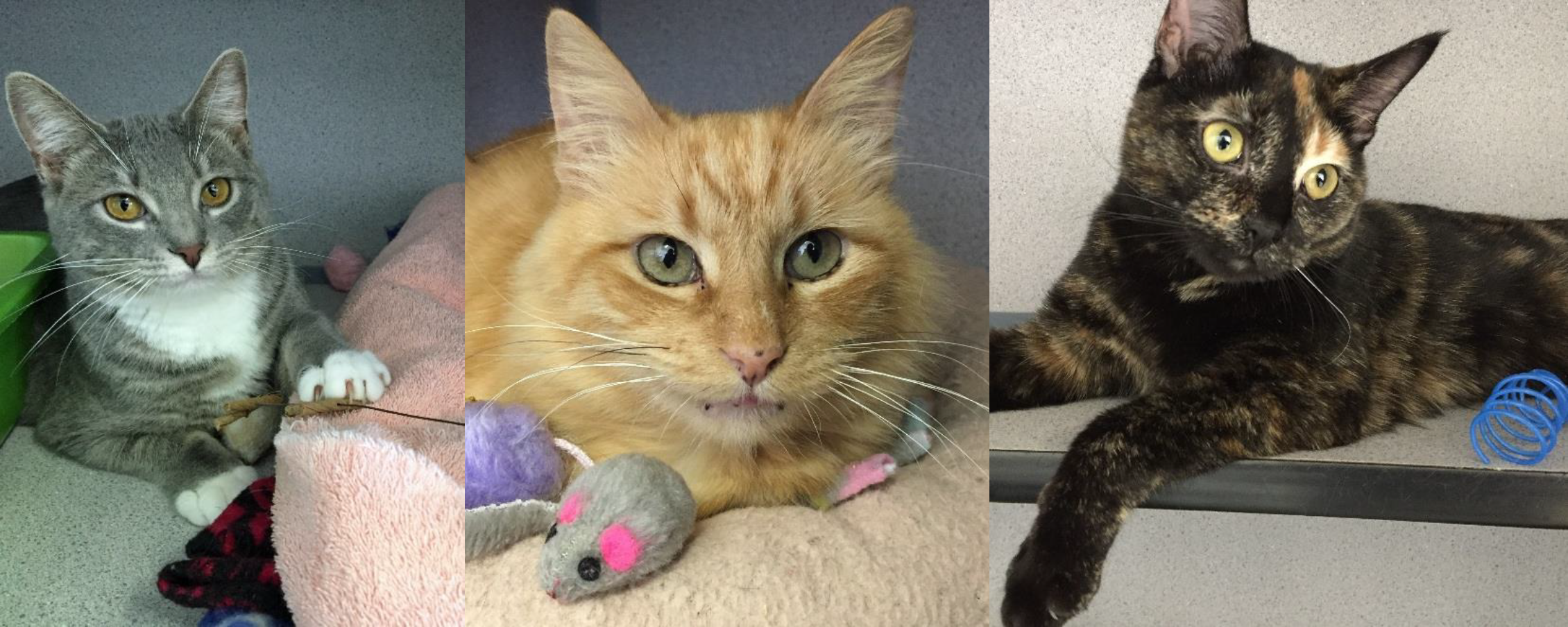

Supplement: Supplementary Figure 1 — Still photographs used in study. [file Image_1.TIF]
